# Factors influencing exclusive breastfeeding among Iranian mothers: A longitudinal population-based study

**DOI:** 10.15171/hpp.2017.07

**Published:** 2016-12-18

**Authors:** Mohsen Saffari, Amir H. Pakpour, Hui Chen

**Affiliations:** ^1^Health Research Center, Baqiyatallah University of Medical Sciences Tehran, Iran; ^2^Social Determinants of Health Research Center (SDH), Qazvin University of Medical Sciences, Qazvin, Iran; ^3^Department of Nursing, School of Health and Welfare, Jönköping University, Jönköping, Sweden; ^4^School of Life Sciences, Faculty of Science, University of Technology Sydney, NSW Australia

**Keywords:** Exclusive breastfeeding, Mothers, Theory of planned behavior

## Abstract

**Background:** Exclusive breastfeeding (EBF) contributes to the health and survival of the newborns. Many factors influence the EBF behavior. This study aimed to identify the determinant factors in order to improve the practice of EBF among Iranian mothers.

**Methods:** A longitudinal study was carried out in 1445 mothers with newborns in Qazvin city, Iran (September 2015-March 2016). Demographic variables as well as the constructs of theory of planned behavior (TBP) were measured by questionnaires. Bivariate analysis using Pearson and Spearman correlation tests with analysis of variance were used to investigate the associations among the variables. Both hierarchal multiple regression and logistic regression were applied to identify potential determinative factors for the EBF.

**Results:** Nearly, 80% (CI: 77.97-82.63%) of the participants had the intention of EBF. All TPB constructs, moral norms, and self-identity were significantly correlated with each other (r: 0.09- 0.40, P < 0.01). Some demographic variables such as age, income, employment and primiparity were also correlated with the EBF (r: 0.11-0.15, P < 0.05). The constructs of the TPB were able to predict the EBF behavior, which account for 49% of the variance in the predicting factors (df = 8, F = 7.70). The self-identity and moral norms accounted for an additional 15% of the variance (df = 10, F = 3.16). Younger mothers with lower socio-economic status were at higher risk of EBF cessation. The intention has a greater impact on the initiation of EBF than perceived behavioral control (PBC) but not for the maintenance of EBF (OR, 2.88 [CI: 2.38-3.48] & 1.13 [CI:1.03- 1.23] vs. OR, 1.27 [CI:1.15-1.39] & 2.66 [CI: 2.02-3.49]).

**Conclusion: ** The interventions to promote knowledge, attitude and behavioral control towards the EBF should be considered especially in the young mothers with low socio-economic status.

## Introduction

Exclusive breastfeeding (EBF) in newborns up to 6 months of age is among the most important public health recommendations to improve children’s health around the world. According to the International Guidelines for Breastfeeding, EBF should be initiated in the first hour of birth and continued during the first 6 months of life exclusively.^1^ It is also recommended to continue breastfeeding with the introduction of solid food for 12-24 months, or as long as the mother and the baby desire.^2^ This can have several advantages for both the mother and the child. For example, previous studies have indicated that in the babies breastfeeding can boost the immune system, lower the morbidity rate due to infectious diseases, improve bone density, benefit mental development, and reduce their risk of being overweight and obese in adulthood.^3-6^ In addition, mothers who breastfed their children, were more likely to return to their pre-gestational body weights soon after the delivery.^7^ Their own risk of developing breast cancer was lower compared to non-breastfeeding mothers.^8^ Moreover, breastfeeding can help to build up a strong bond between the mother and her child, which may help to establish normal or positive feelings and emotions in both mother and child to prevent post-partum depression.^9^

Despite the above advantages of breastfeeding, globally it is estimated that less than 40% of the infants under 6 months received EBF and only 15% may receive continuous breastfeeding along with solids for up to 2 years as per World Health Organization (WHO) recommendations.^2^ In low-income and underdeveloped regions, the situation is even worse. It has been estimated that only 2 in 10 infants may receive appropriate EBF in the first 6 months after the birth.^10^ A recent study in Tanzania reported that only 21% of the newborns received EBF,^11^ and another study in Brazil indicated that only 30% of the infants were breastfed exclusively in the first 6 months.^12^ In addition, a population-based study in Iran showed that only 45% of the Iranian mothers practiced EBF,^13^ while another study indicated that in the regions such as Zahedan, Yazd and Qazvin, the rates of EBF are among the lowest in Iran.^14^

There are various contributing factors that may predict the ongoing practice of EBF and breastfeeding after the initial 6 months after delivery. They can be classified into socio-demographic, psychological, physiological, and interventional factors.^15^ Several studies in different countries have suggested a few potential risk factors for early weaning before 6 months, such as the type of delivery, social-economic status, primiparity, premature pregnancy (teenagers), insufficient break between two gestations, the use of pacifier, and lower maternal education level.^11-13^ However, certain psychological factors may need more attention because of their complexity and culture-based nature to determine breastfeeding behaviors.

The theory of planned behavior (TPB) is commonly used by the psychologists to assess influential factors behind the intention of certain behavior. According to this theory, the behavior may be determined by the intention and self-control to perform such behavior, namely perceived behavioral control (PBC). Indeed, the intention towards performing certain behavior is related to the attitudes, subjective norms, and the PBC.^16^ The TPB has been widely applied to predict many health-related behaviors, including breastfeeding. A study in the United Kingdom showed that the constructs of the TPB may explain 56% of the variance in mother’s intention for breastfeeding.^17^ Similarly, the TPB successfully predicted maternal practice of breastfeeding to complement solid feedings in their children.^18,19^ However, the use of TPB in developing countries especially that to predict the EBF is limited.

Based on a previous study, the constructs such as self-identity and moral norms can be added to the traditional TPB in order to include psychological variables that also contribute to the intention and behavior.^18^ Therefore, we hypothesized that modified model of TPB may provide more comprehensive information than the standard TPB to better predict the behavioral outcomes. Given that previous studies have not applied psychological frameworks to understand the factors associated with breastfeeding especially in developing countries and there is no study on long-term EBF in Iran, a modified TPB along with the other variables will be advantageous. Thus, the current study aimed to use the modified TPB framework to identify the factors determining the practice of EBF, as well as the roles of socio-demographic factors in predicting the long-term breastfeeding behavior among Iranian mothers in a representative population.

## Materials and Methods

### Sample and procedures 

This study was a longitudinal study in pregnant women who were followed up for 6 months after child birth. Data were collected from September 2015 to March 2016 in Qazvin, Iran. There were 1445 women recruited during the third trimester using convenient sampling method. We used an online priori sample size calculator for hierarchical multiple regression analysis^20^ with following parameters: effect size for set B = 0.02, statistical power = 0.99, number of predictors in set A and B = 6 & 7 respectively, and probability level of 0.05.

Maternal care is routinely delivered to all pregnant women by a health care referral system in Iran. There are fourteen health centers in Qazvin city, all of which provide maternal care in their affiliated areas. Clinical information of the pregnant women is normally kept by their health care centers. We invited pregnant women in their last trimester and 91% of them agreed to participate. Their health records were obtained from the health centers. Women who were illiterate and with systemic diseases and breast abnormalities were excluded from the study. Several questionnaires (described below) were used to collect the data and the participants were asked to complete the questionnaires independently. The follow up time was determined based on the dates of baby’s vaccination.

### Measures

**Demographic variables**

Demographic and clinical characteristics of the mothers were collected from their medical records. Age, family income, educational level, employment status, number of children, and obstetric information were included in this study.

**Infant Feeding Knowledge Test Form A**

Prior to the completion of the TPB questionnaire, the mothers were given the definition of the EBF, which reads,* Exclusive breastfeeding is defined as ‘the infant only receiving breast milk without any additional food or drinks, nor water.’*

The mothers’ knowledge on breastfeeding was assessed using a validated method, Infant Feeding Knowledge Test Form A (AFORM).^21^ There are two sections in the AFORM including 10 multiple choices and 10 true-false items. Each true answer has one point and the total score of the correct answers was ranged between 0-20.The AFORM has been translated into Persian and found to be a valid and reliable tool to test mother’s knowledge on breastfeeding,^22^ where the score of the AFORM was positively associated with the drive to initiate breastfeeding in the mothers. The higher the score is, and the greater knowledge on breastfeeding the mother has.

**TPB measures **

All TPB measures in this study were developed based on the guidelines provided by Icek Ajzen to construct a TPB questionnaire.^23^

**Attitude towards breastfeeding**

The attitude towards breastfeeding has been assessed using seven semantic differential scales, such as ‘Breastfeeding a baby would be: ‘unpleasant-pleasant,’ ‘embarrassing-not embarrassing,’ ‘unhealthy-healthy,’ ‘repulsive-attractive,’ ‘inconvenient-convenient,’ ‘unnatural-natural,’ ‘bad-good.’ Responses were assessed using a 5-point scale where the higher scores indicating more positive attitude towards breastfeeding behavior. Negative statements have the lowest score (1) and positive statements have the highest score (5). The Cronbach alpha value was 0.93 and content validity ratio (CVR) using 8 referees was 0.86.

**Subjective norms regarding breastfeeding **

Two items were used to measure the degree of the social pressure from the significant others that can influence the mother’s intention to breastfeed. ‘People who are important to me think that I should breastfeed’ and ‘People who are important to me would approve me breastfeeding my baby.’ These statements were scored from 0 (definitely should not breastfeed/strongly disagree) to 5 (definitely should breastfeed/strongly agree). Total score is ranged from 0 to 10. Cronbach α value and CVR in the present study was 0.89 and 0.82, respectively.

**Perceived behavioral control**

PBC was measured by three items, including “For me, breastfeeding my baby would be…,” ‘difficult’ (1) – ‘easy’ (5); “If I breastfeed my baby, things might get in the way that would stop me from doing it,” ‘unlikely’ (5) – ‘likely’ (1); “How confident are you that you could breastfeed your baby if you want to,” ‘not at all confident’ (1) – ‘very confident’ (5). There was a total score of 3-15 for this scale and higher score indicates a better PBC. There was a high internal consistency in the item scores with a Cronbach α of 0.87 and CVR indicating an acceptable value (0.85, n = 8).

**Intention to breastfeed**

This was measured by five researcher designed items. Example of items were “Do you intend to breastfeed your baby?” or “How strongly do you want to breastfeed?” These items were scored on a 5-point scale ranging from 1 (definitely do not) to 5 (definitely do). Total score is ranged between: 5-25 and higher score shows a stronger intention for breastfeeding. There was adequate internal consistency in the item scores with a Cronbach α of 0.93 and a good content validity (CVR = 0.83, n = 8).

**Moral norms **

Moral norms were assessed by four items commonly used in the breastfeeding domain^24^ including “It would feel right for me to breastfeed my baby,” “I would feel guilty about bottle feeding my baby,” “It would go against my principles to bottle feed my baby,” and “It would feel right for me to bottle feed my baby.” Responses were rated on a 5-point scale from 1 (strongly disagree) to 5 (strongly agree) with a total score ranged between 4 and 20. Higher scores represent greater commitment for moral norms. In this study, there was high internal consistency in the item scores with a Cronbach α of 0.94.

**Self-identity **

Self-identity was measured by two items using a 5-point scale ranged from 1 (strongly disagree) to 5 (strongly agree). The two items were, “Breastfeeding would be an important part of whom I am” and “I would feel upset if I am unable to breastfeed.” There is a total score between 2 and 10, where higher score indicates greater contribution of breastfeeding to self-identity. Internal consistency between the item scores was α = 0.81 and CVR was 0.93 (n = 8).

**Breastfeeding behavior**

Breastfeeding behavior was measured at two time points. At birth, breastfeeding behavior was assessed according to the patient records in the hospitals to investigate if the mothers breastfed their babies soon after the birth. After 6 months, EBF was assessed by a self-reporting method when the mothers were referred to the urban health centers to immunize their children. The breastfeeding behavior was coded as either 0 (none EBF) or 1 (EBF).

### Statistical analysis 

In order to identify potential covariates, a series of analyses were performed. Pearson correlations, Spearman rank correlation, phi correlation and analysis of variance (ANOVA) were performed to investigate demographic and clinical variables that may be associated with breastfeeding behavior. A hierarchical multiple linear regression test was used to assess the predictive power of TPB, moral norms and self-identity as independent variables on the intention to breastfeeding. In Step 1, the independent variables were age, first child, family income, mother’s education, mother’s occupational status, knowledge of breastfeeding, attitude towards breastfeeding, subjective norms and PBC. Self-identity and moral norms were entered into Step 2 of the model. All the independent continuous variables were standardized to reduce multicollinearity among predictor variables as per literature.^25^ Kolmogorov-Smirnov test was performed to check if a variable was normally distributed. Multicollinearity was assessed using the tolerance collinearity statistics and the variance inflation factor (VIF). A tolerance of ≤ 0.20 and a VIF of ≥ 5 indicate a multicollinearity problem.

In addition, two hierarchical logistic regression analyses were conducted to determine which variables can predict mother’s decision to breastfeed. Breastfeeding at both birth and 6 months were considered as a dependent variable for the logistic regression models. In Step 1, the covariates (i.e. age, first child, family income, mother’s education and mother’s occupational status) were entered into the models. In line with the TPB, the intention and PBCs were entered into Step 2 of the analyses. The data were analyzed using SAS 9.2 (SAS Institute, Cary, NC) and *P* < 0.05 was considered significant.

## Results

A considerably high response rate was found in this study (91%). A total of 1445 mothers agreed to participate in the study, where 20 mothers did not return the follow-up questionnaires at 6 months due to reasons such as migration, being busy, baby death and sickness. There was no significant difference in any of the TPB variables or demographic characteristics at baseline between the drop-out mothers and the participating mothers.

All TPB constructs and demographic characteristics are reported in Table 1. Socio-demographic characteristics of the mothers include an average age of 28.6 years (range, 17–44 years) with 6.7 years of education (range, 0–16 years), 75.2% (n = 1086) unemployed, and 52.5% (n = 758) primiparity. The rate of women with the intention to breastfeed was 80.3% (n = 1116, CI: 77.97-82.63). Overall, most of the mothers interviewed in this study had positive attitude and intention towards breastfeeding. They understood the importance of social pressure (subjective norm) to breastfeed their children and claimed that they had control over their practice of breastfeeding.

**Table 1:** Characteristics of the participants

**Variables**	**Time 1 (n = 1445)**
Age (mean ± SD)	28.59 ± 5.46
Years of education	6.70 ± 3.66
Household income (1000 Rials)^a^	918.49 ± 597.36
Knowledge	9.36 ± 3.86
Attitude	4.17 ± 1.33
Subjective norms	3.33 ± 1.35
Perceived behavioural control	3.23 ± 1.31
Behavioral intentions	3.19 ± 1.16
Moral norms	3.35 ± 1.42
Self-Identity	3.28 ± 1.19
Sex of the baby	n (%)
Boy	737 (51.0%)
Girl	708 (49.0%)
First child	
Yes	758 (52.5%)
No	687 (47.5%)
Occupational status	
Employed	359 (24.8%)
Unemployed	1086 (75.2%)
Obstetric data	
Vaginal delivery	494 (34.2%)
Cesarean section	951 (65.8%)
Breastfeeding at birth	
Yes	1161 (80.3%)
No	284 (19.7%)
Breastfeeding at 6 months	
Yes	490 (34.4%)
No	935 (65.6%)

Abbreviation: SD, Standard deviation. ^a^ 35000 Rials = US$1, January 2015.

Bivariate analysis indicates significant associations between age, income, occupational status, as well as primiparity, and the initiation and maintenance of breastfeeding (Table 2). In addition, all TPB constructs, moral norms, and self-identity are significantly correlated with each other (r = 0.17 -0.45, *P* < 0.01).

**Table 2:** Pearson and Phi correlations of the variables

	**2**	**3**	**4**	**5**	**6**	**7**	**8**	**9**	**10**	**11**	**12**	**13**	**14**	**15**
1. Age	.09	-.06	.13^b^	-.21^a^	.05	.10^a^	.08	.06	.08	.15^a^	.07	.05	.16^b^	.08
2. Sex of the baby^c^		.08	.08	.02	.03	.07	.11	.10	.12	.05	.01	.09	.10	.08
3. Occupational status^c^			.42^a^	.03	.21^a^	.16^b^	.04	.01	.09	.16^b^	.04	.09	.16^a^	.11
4. Income				.03	.34^a^	.02	.03	.05	.07	.05	.02	.10	.11^a^	.09
5. First child^c^					-.13^b^	.11	.02	.04	.10	.09^b^	.07	.06	.14^a^	.17^a^
6. Years of education						.12^a^	.09^b^	.10^b^	.09^b^	.11^a^	.05	.08	.19^a^	.16^b^
7. Attitude							.32^a^	.40^a^	.20^b^	.24^a^	.35^a^	.28^a^	.29^a^	.24^a^
8. Subjective norms								.35^a^	.39^a^	.29^a^	.19^a^	.37^a^	.17^a^	.30^a^
9. PBC									.39^a^	.34^a^	.29^a^	.21^a^	.19^a^	.44^a^
10. Intention										.31^a^	.38^a^	.28^a^	.19^a^	.35^a^
11. Breast feeding at 6 months^c^											.27^a^	.26^a^	.29^a^	.48^a^
12. Self-identity												.47^a^	.19^a^	.47^a^
13. Moral norms													.23^a^	.46^a^
14. Knowledge														.28^a^
15. Breast feeding at birth^c^														

Abbreviation: PBC, perceived behavioral control. ^a^ *P* < 0.01; ^b^ *P* < 0.05; ^c^ Phi correlation coefficient.

The predictors of the intention to breastfeed were modeled using hierarchical multiple linear regression. The results were summarized in Table 3. At Step 1, PBC was a highly significant predictor of the intention to breastfeed. The knowledge, attitude and subjective norms also contributed to this prediction. In addition, the analysis indicated that the mothers with higher education and higher household income had stronger intentions to breastfeed. The addition of self-identity and moral norms significantly improved the model by 15% additional variance. Furthermore, after the inclusion of the variables of self-identity and moral norms, all cognitive variables were still significant predictors of the intention to breastfeed. The TPB variables predicted 59% variance in the intentions.

**Table 3:** Hierarchical linear regression of the intention to breastfeed

	**β**	**R** ^ 2 ^ **change**	***F*** ** change**	**SE**	** 95% CI**	
					**Lower**	**Upper**
**Step 1**		0.494	7.703^a^			
Age	0.020			0.031	0.041	0.076
First child						
No	Ref					
Yes	0.041			0.084	-0.124	0.206
Years of education	0.100^a^			0.070	0.034	0.167
Occupational status						
Unemployed	Ref					
Employed	0.067			0.046	-0.240	0.277
Household income	0.109^a^			0.031	0.049	0.169
Knowledge	0.140^a^			0.034	0.073	0.207
Attitude	0.165^a^			0.036	0.094	0.236
Subjective norms	0.322^a^			0.037	0.248	0.395
PBC	0.497^a^			0.035	0.429	0.565
**Step 2**		0.146	3.165^a^			
Age	0.023			0.020	-0.034	0.080
First child						
Yes	Ref					
No	0.015			0.029	-0.043	0.072
Years of education	0.113^a^			0.033	0.047	0.179
Occupational status						
Unemployed	Ref					
Employed	0.062			0.046	-0.152	0.028
Household income	0.167^a^			0.052	0.064	0.269
Knowledge	0.103^a^			0.034	0.036	0.170
Attitude	0.102^a^			0.037	0.028	0.175
Subjective norms	0.289^a^			0.038	0.216	0.362
PBC	0.384^a^			0.040	0.305	0.463
Self-identity	0.215^a^			0.043	0.131	0.299
Moral Norms	0.077^b^			0.034	0.010	0.144

*R* ^2^= 0.640, adjusted *R*^2^= 589; ^a^*P* < 0.01; ^b^*P* < 0.05. Abbreviations: PBC, perceived behavioral control; SE, standard error; Ref, reference group.

Two hierarchical multiple logistic regressions were used to test the predictive validity of the TPB (i.e. intention and PBC), in relation to the initiation and maintenance of breastfeeding (Table 4). Mothers who were unemployed or housewives, or had a higher educational status were more likely to breastfeed their children at birth in Step 1. In Step 2, the PBC and the intention significantly predicted the initiation of breastfeeding. The power of the intention was greater than the PBC to predict the initiation of breastfeeding (Table 4).

**Table 4:** Binary logistic regression of the initiation and maintenance of breastfeeding‏

**Variable**	**Breastfeeding**					
	**Initiation**			**Maintenance**		
	**OR**	**95% CI**		**OR**	**95% CI**	
		**Lower**	**Upper**		**Lower**	**Upper**
**Step 1**						
Age	0.986	0.960	1.012	0.984	0.959	1.010
First child						
No						
Yes	1.346	0.997	1.817	1.347	1.011	1.795
Years of education	1.121	1.081	1.162	1.115	1.076	1.156
Occupational status						
Employed						
Unemployed	2.233	1.509	3.304	1.923	1.291	2.862
Household income	0.977	0.945	1.011	0.991	0.959	1.024
**Step 2**						
Age	0.985	0.957	1.013	0.986	0.961	1.011
First child						
Yes						
No	1.321	0.960	1.820	1.347	1.004	1.807
Years of education	1.090	1.047	1.134	1.103	1.063	1.145
Occupational status						
Employed						
Unemployed	2.109	1.372	3.240	1.681	1.113	2.539
Household income	1.081	1.030	1.134	0.993	0.960	1.027
Intention	2.881	2.382	3.484	1.130	1.036	1.232
PBC	1.272	1.158	1.398	2.663	2.028	3.497

Abbreviations: PBC, perceived behavioral control; OR, odds ratio. ^a^ *P *< 0.05; ^b^*P *< 0.01; ^c^*P *< 0.001.

For the maintenance of breastfeeding at 6 months, the analysis indicated that the mothers who were unemployed or housewives, had a higher education, or were primiparous were less likely to continue EBF at 6 months. The inclusion of the intention and the PBC improved the predictive validity of the model. However, different from the initiation of breastfeeding, the contribution of the PBC to predict breastfeeding maintenance was greater than that of the intention (Table 4).

## Discussion

This study aimed to investigate the sustainability of the EBF among Iranians mothers and identify the contributing factors to the EBF behaviors especially those related to the TPB. Although, nearly 80% of our participants claimed to have the intention for breastfeeding after child delivery, only 34% have reported the ability to maintain such behavior at 6 months. Cognitive variables such as the knowledge of the beneficial effects of breastfeeding, the attitude, subjective norms, self-identity, and moral norms are the significant factors to predict both the intention and the practice of the EBF.

The insufficient practice of EBF has been shown to be a significant health issue in several previous studies. Despite the use of a cross-sectional design, the majority of those previous studies reported a low rate (50%) of EBF at 6 months after delivery.^11-14^ For example, in a study in Bangladesh only 36% of 6 months old infants received EBF.^26^ Similar finding has been reported in southeast Asia, where the EBF rates were decreased at 3, 4 and 6 months post-delivery (48%, 26% and 11% respectively).^27^ Low rates of EBF seem to be prominent particularly in under developed countries, while the EBF is better practiced in developed countries. McDonald et al estimated that nearly 62% of a Canadian population had practiced EBF as per recommendation by the WHO.^28^ Similarly, Nielsen and colleagues showed that in Scotland the percentage of infants receiving breastfeeding at 15 and 25 weeks of age was higher than the world average.^29^

According to the behavioral paradigms, most healthy behaviors are difficult to maintain. However, it is suggested that when someone has strong intention of certain behavior, the initiation of such behavior is more likely to happen than those with poor intentions.^16^ In the current study, of those who had the intention for EBF at birth, only less than half could still continue EBF after 6 months. This indicates that the intention towards a behavior, as a predicting factor, is not adequate to maintain the behavior; while many other factors also contribute. Considerable number of these factors are cognitive based and may be included in the theories of behavior change, such as the TPB. In the study by Bai et al using the TPB, the constructs such as attitude, subjective norm, and PBC have been demonstrated to be significantly associated with the intention towards the EBF.^30^ Similarly, in another study, the TPB constructs have been shown to play a noticeable role in predicting the EBF behavior for 6 months and could explain nearly 50% of the variance of the intention.^31^ These findings are comparable to our results in this study, as we also found significant correlation between all components of the TPB and EBF intention, as well as a 59% variance of intention which can be explained by the TPB variables.

The main constructs of PBC and intention played considerable roles in predicting EBF in the current study. Both these constructs were significant in the initiation of the EBF, while the intention has a higher predictability than the PBC constructs. However, at 6 months, the power of prediction is stronger with the PBC. This finding suggests that although the intention for EBF may be more important than the PBC for the mothers to make decision on EBF, the ability to continue EBF is highly dependent on the PBC. In another words, if the mothers decide to practice EBF without adequate confidence and control of their behavior, they may not act according to their intentions.

As expected, using the modified model of TPB, the predictability was improved markedly, by a further 15% increase after including the new constructs (social identity and moral norms). As Abrams and Hogg have suggested, behavior-intention association may be more likely to be enhanced when a positive social identity is presented.^32^ Similarly in our study, moral norms (i.e., the perception of moral correctness of a behavior) have been recognized to have a direct effect on the behavior by closing the gap between the intention and behavior. Positive results of using these new constructs in the TPB have also been reported previously.^18^

Although TPB has been recognized by previous studies as a reliable and valid theory to predict healthy behaviors, there may be opportunities to enhance its prediction power with additional psychological factors, such as self-identity and moral norms. Indeed, self-identity may have an additive role in PBC because a person would be more aware of the control of certain behavior when it is self-oriented. Additionally, moral norms may have a direct impact on the intention toward certain behavior. In a simple word, we are more likely do things well accepted by the society which is the value making us socially good persons.

In the present study, in addition to several psychological variables, a number of socio-demographic characteristics were also assessed. We found that the variables, such as age, economic status and employment, may also be associated with the intention. Furthermore, the education level and income have also been identified to be significant predictors. Similar findings have been reported in previous studies. For instance, it has been found that maternal age less than 20 years can increase the risk of early weaning by 6 times.^33^ Lower maternal education level has been identified as a potential risk factor to interrupt the EBF.^34^ The mothers, who are employed, would have inadequate time for baby care and usually are separated from their children during working hours; therefore, working commitment can disrupt EBF.^35^ High household income is a positive determinant of the EBF, which may also be justified by the unemployment status. This is because that in the families with adequate amount of income, it is not necessary for the mothers to work, who thus can have sufficient time to breastfeed their infants.

Primiparity was also a determinant to prevent EBF in our study. This has also been previously reported in India.^33^ The potential explanation may be related to insufficient knowledge and skills for proper breastfeeding, as well as the misperception that the EBF may have unfavorable effects on the physical fitness and postpartum recovery.

We also need to acknowledge several limitations in this study. Firstly, we recruited the participants from a city that may not represent the entire Iranian populations because of the cultural difference in the other regions. However, we have recruited sufficient number of mothers and the follow-up rate was also considerably high which increased the internal validity of our findings. Secondly, the practice of EBF has been measured by self-reporting where the information biases may be problematic. However, there is no objective measure available for a study of this type. Thirdly, other psychological variables, such as self-efficacy, self-concept and social support, may also significantly contribute to the intention and maintenance of EBF, which can be included in the future studies. Fourthly, we used regression analysis to investigate the association between the variables, whereas more sophisticated method such as path analysis or structural equation modeling may provide additional information on mediator or moderating factors. This can be considered in our further studies. Lastly, we examined the EBF at 6 months after delivery, and future studies can extend the end point to 2 years as per WHO recommendation.

## Conclusion

Both socio-demographic and psychological variables can significantly affect the practice of EBF. However, subjective variables, such as behavioral control, subjective norms, knowledge, and attitude, may be stronger indicators of breastfeeding than the socio-demographic variables. Recognizing these variables may help to develop strategies to overcome the potential barriers to improve the rates of breastfeeding in the mothers of certain socio-demographic group, such as younger mothers with poor education or income. Furthermore, the TPB may be a useful theoretical framework to measure the determinant factors of the intention toward the EBF in future studies.

## Ethical approval

This study (registered ID 78598-AC) was approved by the Human Research Ethics Committee of the Qazvin University of Medical Sciences, Qazvin, Iran. All participants signed the consent form and confidentiality of data collection was ensured to all of them.

## Competing interests

Nothing to declare.

## Authors’ contributions

MS and AHP designed the study and wrote the manuscript. AHP collected all the data. Data were analyzed by MS and AHP. HC contributed to the writing of the manuscript.

## Acknowledgments

The authors would like to thank all the staff in Qazvin Health Centers who contributed in data collection.
